# Single-cell profiling indicates a high similarity between immune cells in the cerebrospinal fluid and in meningeal ectopic lymphoid tissue in experimental autoimmune encephalomyelitis

**DOI:** 10.3389/fimmu.2024.1400641

**Published:** 2024-06-12

**Authors:** Tanya Georgieva, Jolien Diddens, Verena Friedrich, Gildas Lepennetier, Rosa Margareta Brand, Klaus Lehmann-Horn

**Affiliations:** Department of Neurology, TUM School of Medicine and Health, Technical University of Munich, Munich, Germany

**Keywords:** multiple sclerosis, single-cell sequencing, experimental autoimmune encephalomyelitis, B cells, anti-CD20 treatment, ectopic lymphoid tissue

## Abstract

**Background and objectives:**

B cell depleting anti-CD20 monoclonal antibodies (aCD20 mAbs) are highly effective in treatment of multiple sclerosis (MS) but fail to halt the formation of meningeal ectopic lymphoid tissue (mELT) in the murine model experimental autoimmune encephalomyelitis (EAE). While mELT can be examined in EAE, it is not accessible *in vivo* in MS patients. Our key objectives were to compare the immune cells in cerebrospinal fluid (CSF), which is accessible in patients, with those in mELT, and to study the effects of aCD20 mAbs on CSF and mELT in EAE.

**Methods:**

Applying single cell RNA sequencing, we compared gene expression profiles in immune cells from (1) CSF with mELT and (2) aCD20 mAbs treated with control treated mice in a spontaneous 2D2xTh EAE model.

**Results:**

The immune cell composition in CSF and mELT was very similar. Gene expression profiles and pathway enrichment analysis revealed no striking differences between the two compartments. aCD20 mAbs led not only to a virtually complete depletion of B cells in the CSF but also to a reduction of naïve CD4+ T cells and marked increase of macrophages. No remarkable differences in regulated genes or pathways were observed.

**Discussion:**

Our results suggest that immune cells in the CSF may serve as a surrogate for mELT in EAE. Future studies are required to confirm this in MS patients. The observed increase of macrophages in B cell depleted CSF is a novel finding and requires verification in CSF of aCD20 mAbs treated MS patients. Due to unresolved technical challenges, we were unable to study the effects of aCD20 mAbs on mELT. This should be addressed in future studies.

## Introduction

Anti-CD20 monoclonal antibodies (aCD20 mAbs), depleting B cells, are very efficient in treatment of relapsing-remitting forms of multiple sclerosis (MS) (1, 2). Studies have also shown moderate beneficial effects in treatment of progressive MS (PMS), particularly in progression independent of relapse activity (PIRA) in early relapsing-remitting MS and in active primary PMS (3, 4). B cell-rich inflammatory cellular infiltrates in the meninges have been observed in patients with MS and in its murine model, experimental autoimmune encephalomyelitis (EAE). We designated these structures as meningeal ectopic lymphoid tissue (mELT). The presence of mELT in MS patients correlates with an earlier disease onset, a more rapid disease progression, and subpial cortical damage (5–10). mELT resembles secondary lymphoid organs (SLO) (11) to varying degrees and previous studies suggest that it may have similar functions, particularly germinal center activity (12–14). Interestingly, the B cells compartments in peripheral blood and CSF of MS patients are clonally related and exchange across the blood brain barrier (BBB) (15).

Based on the paradigm that 1) mELT contributes to progression in MS by facilitating smoldering inflammation in the central nervous system (CNS) behind the BBB and 2) assuming that B cells are an integral and pivotal component of mELT, we investigated the effects of aCD20 mAbs on mELT in a previous study (16). Applying murine aCD20 mAbs in a spontaneous chronic EAE model that has myelin oligodendrocyte glycoprotein (MOG) specific B and T cells (2D2/TCRMOG × Th/IgHMOG mice) and features mELT in the spinal cord (17, 18), we could confirm previous findings that aCD20 mAbs do not alter the disease course in this particular model (19). One possible explanation for this is, that large amounts of MOG-binding antibodies, which are present even after sustained B cell depletion, in collaboration with TCRMOG 2D2 T cells, are sufficient to induce and maintain EAE. Like in a case in which rituximab depleted B cells from cerebral perivascular spaces (20), we could demonstrate in our previous study that aCD20 mAbs depleted virtually all B cells from the meningeal compartment in EAE. The surprising finding, however, was that mELT forms anyway, despite the absence of B cells to a comparable extent (16). This raises important questions regarding the function of mELT in CNS autoimmunity and whether mELT even without B cells may have a role in MS. If mELT devoid of B cells still contributes to EAE pathogenesis, this may provide an alternative explanation why B cell depleted EAE mice develop EAE like B cell competent control animals and, potentially, why we observe disease progression in MS patients treated with aCD20 mAbs. Thus, a primary objective of this study was to address potential differences and common features of mELT with or without B cells. To address this question, the 2D2xTh EAE model is suitable, even though it does not respond to B cell depletion clinically.

Studying mELT functionally relies predominantly on experimental models. Extracting or otherwise accessing mELT in MS patients is challenging and can be reasonably achieved only in post-mortem tissue. Since cerebrospinal fluid (CSF) is in a spatial relationship with the meningeal compartment and can be readily and routinely obtained in patients, the second objective was to examine similarities and differences between the mELT and CSF compartments in respect to their immune cell composition and phenotype.

In the present study, we applied single cell RNA sequencing to investigate how B cell depletion by aCD20 mAb changes the cellular composition and immune cell phenotypes in mELT and CSF. We here fore used the same murine EAE model introduced above. We compared the immune cell composition in mELT and CSF in B cell competent conditions. Due to technical challenges, which precluded us from obtaining sufficient data from one experimental condition, the aCD20 mAb-treated mELT compartment, we could only approach our primary objective indirectly. After confirming that the immune profile of CSF and mELT in B cell competent mice was quite similar, we used the CSF compartment as a surrogate for mELT when examining the effects of aCD20 mAbs on the meningeal compartment. Our study revealed that the cellular composition and gene expression profiles of mELT and CSF are very similar. aCD20 mAbs efficiently depleted CSF B cells and concomitantly led to a reduction of naïve CD4+ T cells and increase of macrophages.

## Materials and methods

### 2D2×Th mice

2D2xTh EAE mice – which develop spontaneous chronic EAE and meningeal inflammatory lesions in the spinal cord - were generated, held and clinically assessed as previously described (16–18). All animal procedures were approved by the standing committee for experimentation with laboratory animals of the administration of Upper Bavaria (ROB-55.2-2532.Vet_02-16-100).

### Anti-CD20 treatment and B cell depletion

**Figure 1:**
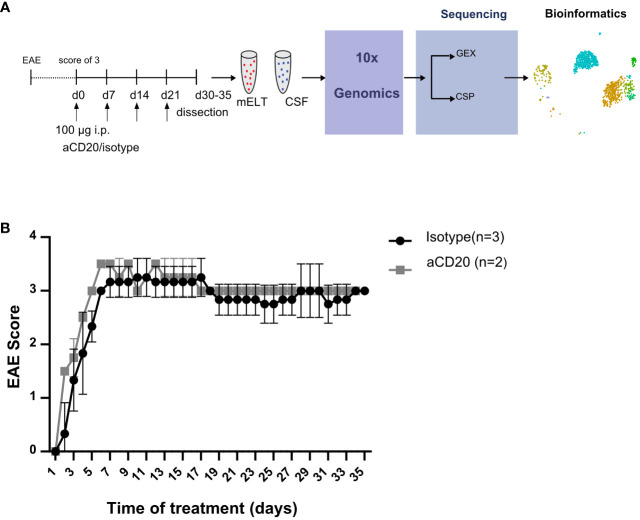
Study design and EAE score. **(A)** Mice were treated with a weekly dose of 100 μg of aCD20 mAb (n = 2) or isotype control (n = 3). On day 30-35, mice were dissected, and cell surface protein (CSP) and gene expression (GEX) libraries prepared and sequenced using 10x Genomics technology before bioinformatics analyses. **(B)** Daily evaluated EAE scores over the experimental period. Error bars show +/- SEM for the control group only.

Six mice were initially used in the current experiment. Mice were randomly assigned to either the treatment or control group. The treatment was initiated when a mouse reached an EAE score ≥ 3 and was conducted as previously described (16). In brief, B cell depletion was achieved by weekly injection of 100 μg murine aCD20 mAbs (clone 18B12, provided by Roche). *In vivo* grade mouse IgG2a kappa isotype control (Crown bio, Cat: C0006) was used in the control group. One mouse from the treated group was excluded from further analysis due to insufficient effect of the aCD20 treatment. **Figure 1A** shows the experimental design. **Table 1** lists the clinical characteristics of the 5 mice included in the final analyses and **Figure 1B** their clinical disease course.

**Table 1:** Characteristics of all mice utilized in the experiment.

Sex	treatment	Age at sacrifice (days)	Tissues analyzed	Age at disease onset (days)	disease duration (days)	Max. EAE Score	Mean EAE score	EAE score at sacrifice
female	aCD20	66	mELT, CSF	32	34	3	2,9	2,5
female	aCD20	77	mELT, CSF	46	31	3	2,6	3
female	Isotype	64	mELT, CSF	32	32	3,5	3,1	3
female	Isotype	61	mELT, CSF	31	30	3	2,7	2,5
male	Isotype	66	mELT, CSF	31	35	3	3	3

### Sample collection and single-cell preparation

Mice from both groups were sacrificed 30-35 days after the first injection. Animals were first anesthetized, after which CSF was obtained by puncture of the cisterna magna and stored on ice. Subsequently mice were sacrificed. After thorough perfusion with ice cold PBS, the spinal cord was dissected. A small test piece was cut from the thoracolumbar part of the spinal cord, which was used to histologically confirm the presence of mELT. Meninges were gently separated from the spinal cord in a petri dish with PBS and, using fine forceps, cut into small pieces under a dissection microscope. Subsequently meninges were digested in 600 µl digestion mix consisting of 4µl DNase, 60µl Collagenase IV and 536µl RPMI Medium for 30 minutes at 37°C on a shaker. Cells were isolated from the digested meninges by passing through 30 µl cell strainers, and washed. Cells from meninges and CSF were finally centrifuged (450xg, 5 min, 4 degrees) and resuspended in 100 µl FACS Buffer.

A brief incubation (10 min, 4°C) with Fc Block reagent was performed to block nonspecific antibody binding. Next, samples were labelled with DNA barcoded TotalSeq antibodies against mouse CD4, CD8a, CD19, Ly6G, and NK1.1, as previously described (11). At the same time, samples were incubated with 2 different TotalSeq Hashtag antibodies, in order to allow pooling samples per two: CSF und meningeal cells were incubated (20 minutes at 4° in the dark) with TotalSeq-C 0301 anti-mouse #1 (clone M1/42; 30-F11, Biolegend, dilution 1:400 in FACS buffer) and TotalSeq anti-mouse 0302 #2 (clone M1/42;30-F11, Biolegend, dilution 1:400 in FACS buffer), respectively. The incubation was followed by 3 wash steps with FACS Buffer and cell counting (Cellometer Auto 2000, Nexcelom Bioscience). Ultimately, the cell concentration was adjusted to 1x10^3^ cells/µl in RPMI/10% FCS, and CSF and meningeal cells were pooled together in a 1:4 ratio per mouse.

### Library preparation and sequencing

Cells were loaded onto 10x Genomics Chromium Next GEM Chip G and the Chromium Controller was run according to the manufacturer’s instructions (10x Genomics, USA) with a targeted cell recovery of 10,000 cells. Library preparation of Cell Surface Protein Library (CSP Library) and Gene Expression Library (GEX Library) was performed using Chromium Next GEM Single Cell V(D)J Reagent Kits v1.1, following the 10x Genomics protocol CG000208. The quality of library preparation was assessed using the Agilent 2100 Bioanalyzer.

### Library quantification and single cell RNA-seq

Real-Time PCR was performed to quantify the libraries. All single cell gene expression, BCR and cell surface protein (CSP) libraries were multiplexed and sequenced using Illumina NovaSeq 6000 (S2 Flow cell), to obtain 26 x 91 bp paired-end reads for gene expression, cell surface protein and BCR libraries, taking into account the differences in read depth requirements. Novaseq sequencing was performed at the Helmholtz Zentrum München (HMGU) by the Genomics Core Facility.

### Bioinformatical analysis

#### Single cell data processing and batch correction

Data processing and batch correction were done on all 5 samples as described before (11). In order to exclude dead/dying cells and reduce potential doublets, we kept only cells with >200 and <2,000 genes, >100 and <10,000 read count, <5% of reads mapping to mitochondrial genes and hemoglobin genes. 33.9% of the cells were removed by this quality filtering to obtain 17,226 cells at the end.

#### Cluster annotation

Gene markers for each cluster were defined using FindAllMarkers function (Seurat R library) (v 4.0.0). Each cluster was annotated automatically using CIPR (version 0.1.0) based on the “immgen” database. In order to confirm cluster annotation and, detect specific cell types that did not exist in the database, we used Nebulosa (version 1.0.0) to display gene expression density for several gene markers. Plasma cells could not be detected by the annotation of the clusters using CIPR, we therefore used the *Sdc1* and *Tnfrsf17* gene markers to annotate those cells. Glial cells and CD8+ T cells were annotated using the same approach using the *Slc1a2* gene and *Cd8a* gene respectively. After extracting the gene markers for each cluster and inspecting them visually, we decided to name the “Pre-T cell” group “T cell”, as it fits better the biological reality.

#### Cell surface protein data processing and doublet filtering

TotalSeq antibody barcodes were processed in 2 separate assays: one for the tissue of origin and one for the cell type. We used the centred log-ratio (CLR) normalization method, then the HTODemux function (Seurat) was used to label each cell. Unfortunately, hashtag oligo (HTO), indicating the tissue of origin, for mELT had a relatively lower expression and yielded only 5% of the total cells, as compared to 31% cells labeled as CSF. In addition, 54% of the cells were HTO-negative. To improve the yield and differentiate between tissues, we used a 1.5-fold difference in HTO expression per cell. This approach allowed us to rescue mELT cells not labeled because of the low expression of the mELT tag compared to the CSF tag. Using this approach, we could label 44% of cells as CSF origin and 33% as mELT (77% HTO-positive), with 23% still unknown (HTO-negative) for a total of 17,226 cells. The HTO-negative cells were removed.

The cell type specific CSP antibody barcodes defined by HTODemux yielded 3% CD19, 17% CD4, 2% CD8a, 2% Ly6G, 2% NK, 60% unknown (putative doublet) and 14% negative cells. We did not filter out these putative doublets, because of the low confidence in the CSP-data, instead we used the DoubletFinder R package (v2.0.3) to identify doublets that were not filtered in the previous steps based on the gene expression.

We used the CSP information to remove cells that were confidently labelled as one cell type but clustering with a different cell cluster (Example: cells tagged with the antibody for NK cells but found in the B cell cluster). In addition, we also used the BCR-sequencing information to remove cells having B cell information but not in the plasma cell or B cell cluster. Contaminating glia cells, oligodendrocytes and erythroid cells were removed. The remaining 9,585 cells, annotated in 11 cell types, were used for the differential gene expression analysis, that was performed when a condition had at least 50 cells.

#### Differential gene expression

Differential gene expression was analyzed as described before (11). Clusters comprising fewer than 50 cells were excluded from the analysis. Significant genes (adjusted p-value <0.05) were ranked based on their average log2FC values. The genes mentioned in the text are the 10 most upregulated and 10 most downregulated genes in both comparisons (CSF aCD20 vs CSF isotype and mELT isotype vs CSF isotype).

#### Pathway enrichment analysis

Using the web-based analytical resource Metascape, we identified significant differences in biological pathways between mELT and CSF, as well as between aCD20-treated and untreated mice (21). We utilized the December 18, 2021 version of Metascape and employed mouse data as input, which was analyzed as if it were human data. The following pathway types were chosen for analysis: GO-Biological processes, WikiPathways, Reactome, and KEGG Pathways. Enrichment analysis was conducted against a custom background gene list, containing all genes considered in the differential gene expression analysis. Pathways with an adjusted p-value <0.001 were considered. Ribosomal genes were excluded from the analysis.

## Results

### Immune cells in CSF and mELT form 11 clusters

**Figure 2:**
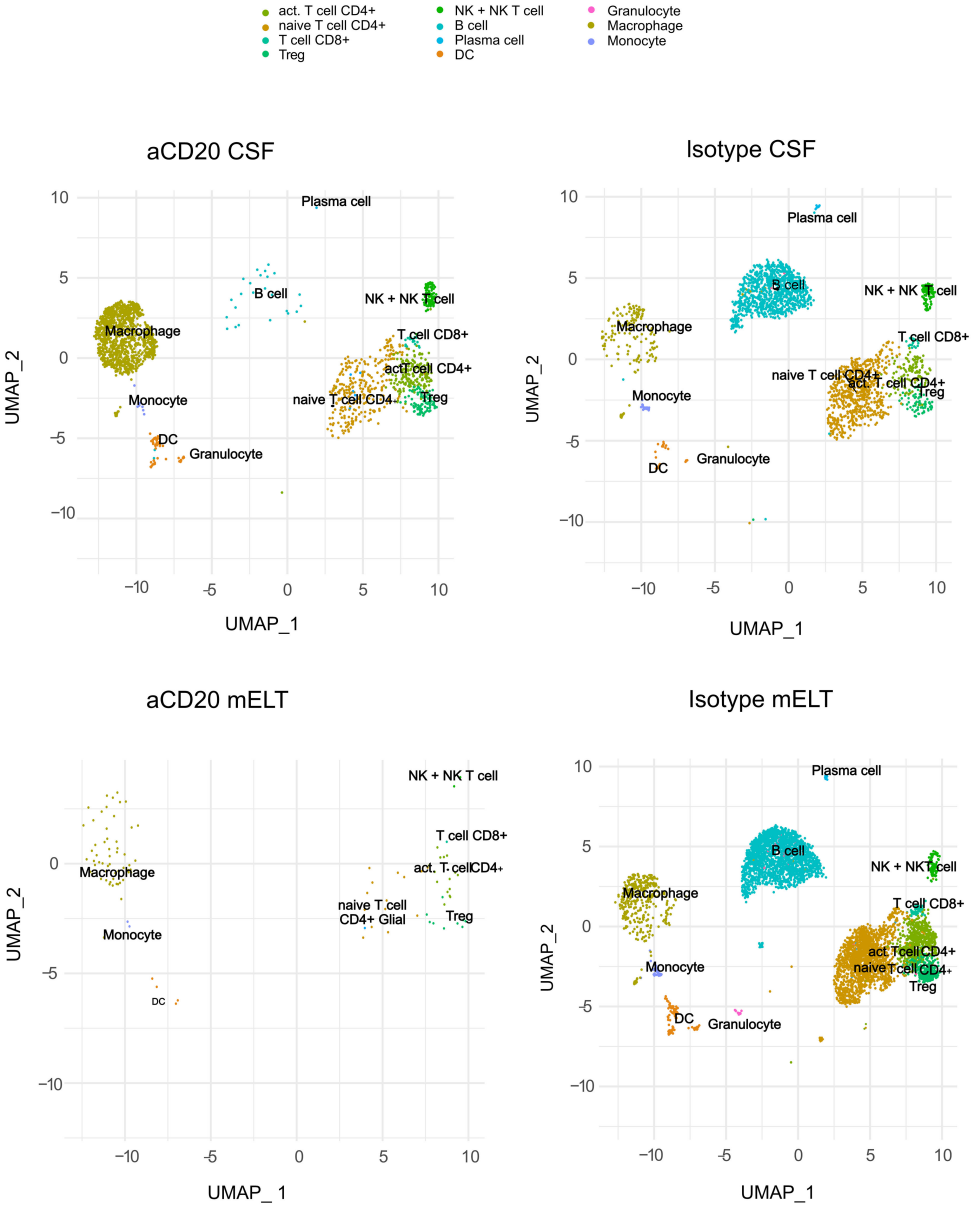
Uniform manifold approximation and projection (UMAP) plot representing 11 color-coded cell clusters identified in using single cell sequencing, split per tissue (CSF and mELT) and per treatment (aCD20 treated mice and control treated mice).

We aimed to examine and compare the immune cell composition of CSF and mELT. Furthermore, we aimed to analyze the cellular differences in these tissues between the aCD20 treated (B cell depleted) and the control treated (B cell competent) mice. After filtering out low quality cells and corrections using “harmony” library, we obtained the transcriptome from a total of 9,585 single cells: 5,445 cells from mELT and 4,140 cells from CSF. We performed clustering and, in this way, classified the cells into 11 final cell clusters (**Figure 2**). Based on a combination of automatic cluster annotation (CIPR version 0.1.0, “immgen” database) and marker gene expression (see Methods section), we identified the following 11 cell types: T cells (*Cd3e*), more specifically naïve CD4+ T cells (*Cd4*, *Ccr7, Sell*), activated CD4+ T cells (*Cd4*, *Rora, Pdcd1, Il2*), CD8+ T cells (*Cd8a*), and regulatory T cells (*Foxp3*), B cells (*Cd79a, Cd19*) and plasma cells (*Sdc1, Tnfrsf17*), a cluster containing natural killer (NK) cells and NKT cells (*Klrb1c, Klrk1*), myeloid lineage cells, separated into dendritic cells (DC) (*Flt3*), macrophages (*Cd68*, *Adgre1*), monocytes (*Cd68*), and granulocytes (*S100a8* and *S100a9*). Erythroid cells (*Hbb-bt*) and oligodendrocytes (*Olig1*), probably coming from blood or CNS contamination respectively, were not considered in further analyses.

### mELT and CSF have a similar cellular composition

**Figure 3:**
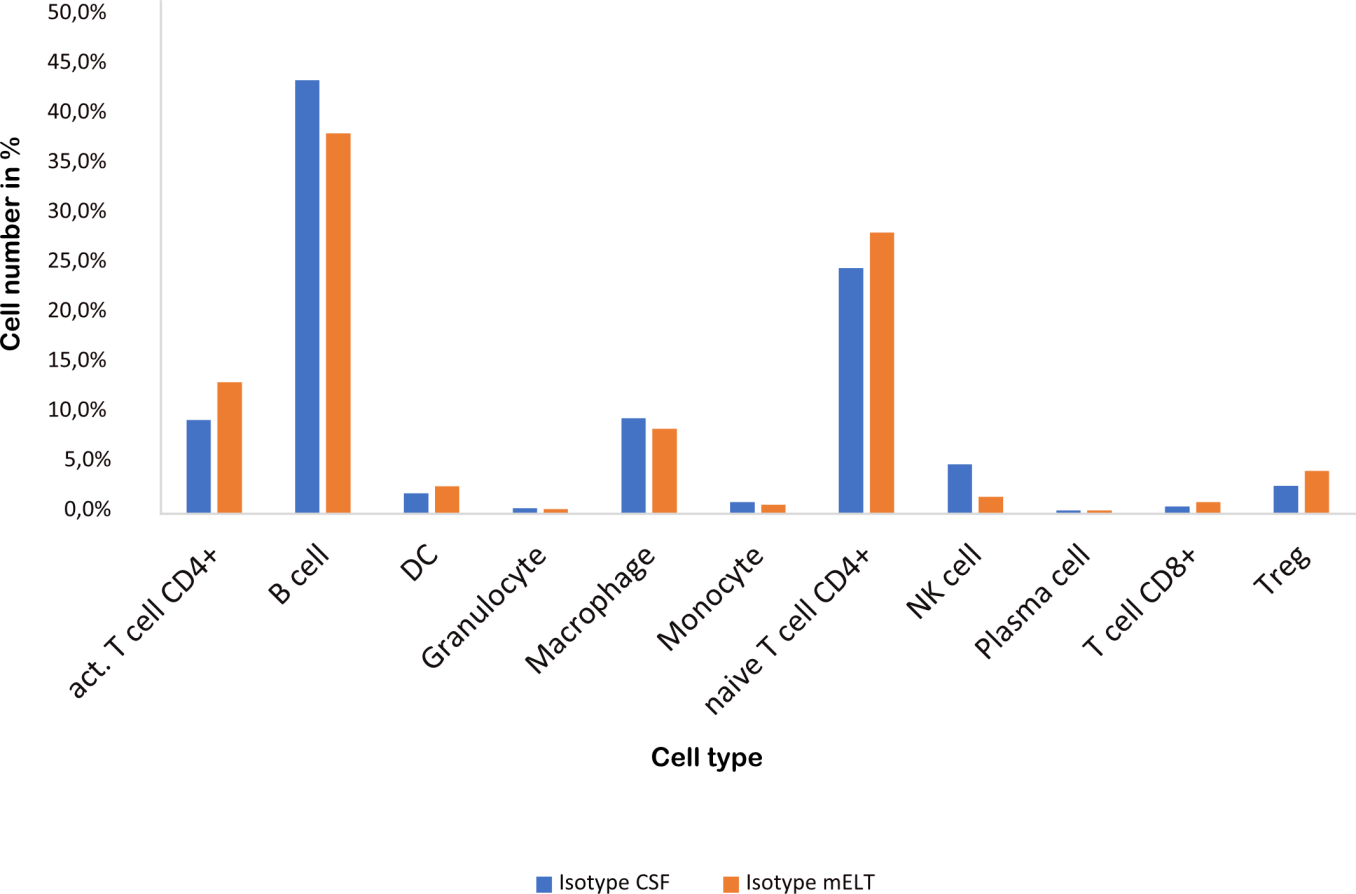
Cell numbers in CSF from control treated mice vs. mELT in control treated mice are similar. The percentage of the respective cell types is shown.

**Figure 3** and **Table 2** illustrate the cell composition of mELT and CSF from control treated (B cell competent) mice. Both CSF and mELT consist mainly of B and CD4+ T lymphocytes. Their relative numbers (percentage) were comparable in both tissues (**Table 2**). Other cell types like monocytes, plasma cells, NK cells, macrophages and granulocytes are also equally represented in both CSF and mELT from control treated mice. Overall, regarding the immune cell composition, CSF and mELT were closely related in control treated 2D2xTh EAE mice.

**Table 2:** Cell composition per tissue.

Cell Type	aCD20 CSF	aCD20 mELT	Isotype CSF	Isotype mELT
**Activated CD4+ T cell**	243 (11,9%)	19 (17,9%)	223 (9,4%)	784 (13,3%)
**B cell**	27 (1,3%)	0 (0%)	1031 (43,6%)	2266 (38,3%)
**DC**	52 (2,6%)	5 (4,7%)	49 (2,1%)	163 (2,8%)
**Granulocyte**	1 (0,01%)	0 (0%)	13 (0,6%)	27 (0,5%)
**Macrophage**	1277 (62,5%)	57 (53,8%)	227 (9,6%)	507 (8,6%)
**Monocyte**	7 (0,3%)	2 (1,9%)	28 (1,2%)	53 (0,9%)
**Naive CD4+ T cell**	230 (11,3%)	12 (11,3%)	584 (24,7%)	1674 (28,3%)
**NK cell**	103 (5%)	2 (1,9%)	118 (5%)	101 (1,7%)
**Plasma cell**	1 (0,1%)	0 (0%)	8 (0,3%)	21 (0,4%)
**CD8+ T cell**	20 (1%)	1 (0,9%)	17 (0,7%)	69 (1,2%)
**Treg**	82 (4%)	8 (7,6%)	66 (2,8%)	254 (4,3%)

Total number of cells (over all mice), as well as the percentage of cells per cell type are given for each tissue.

### CSF from aCD20 treated mice contains less B cells and naïve CD4+ T cells but more macrophages

**Figure 4:**
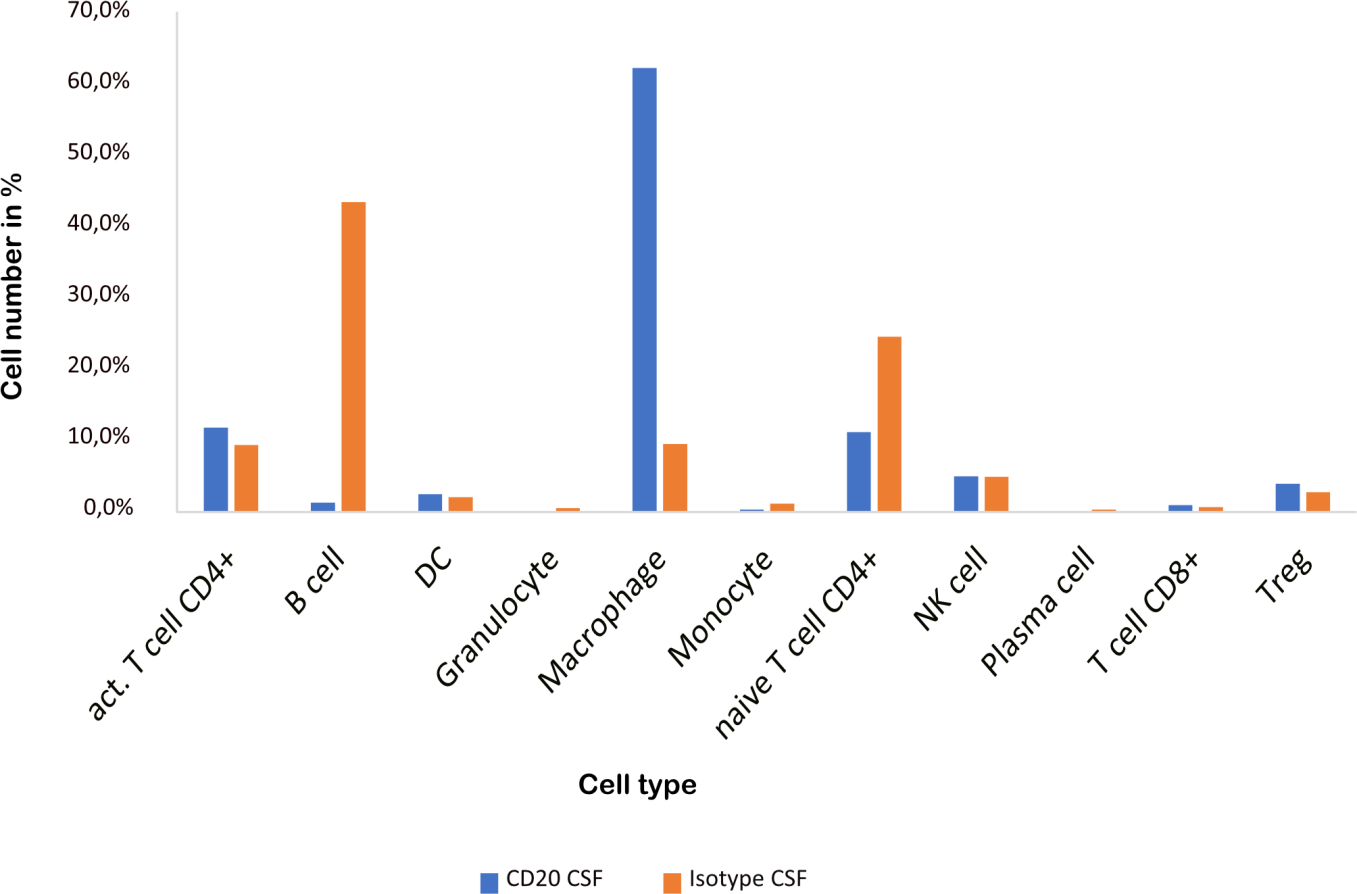
CSF from aCD20 treated mice has less B cells and naïve CD4+ T cells, but more macrophages than CSF from control treated mice. The percentage of the respective cell types is shown.

As previously shown (16), aCD20 treatment did not alter the EAE course (**Figure 1B**). The cellular composition of B cell depleted, aCD20 treated CSF seems to be considerably different compared to the B cell competent control group. Due to insufficient data for the mELT aCD20 condition (**Table 2**), we compared CSF from aCD20 treated versus control treated mice as a surrogate (**Figure 4**, **Table 2**). Like previously shown for mELT (16), aCD20 treatment depleted virtually all B cells from CSF. In addition, aCD20 treated CSF contained less naïve CD4+ T-cells. Similarly, we observed a reduction of monocytes, granulocytes, and plasma cells, but their number was relatively low overall. Interestingly, we observed a substantial increase of macrophages in CSF samples from the aCD20 treated group when compared to the control treated group (1,277 vs. 227 cells; 62,5% vs. 9,6%). Numbers of activated CD4+ T cells, NK cells, Tregs, CD8+ T cells were similar in both conditions.

### aCD20 treatment results in heterogeneous up- and downregulation of proinflammatory genes

**Figure 5:**
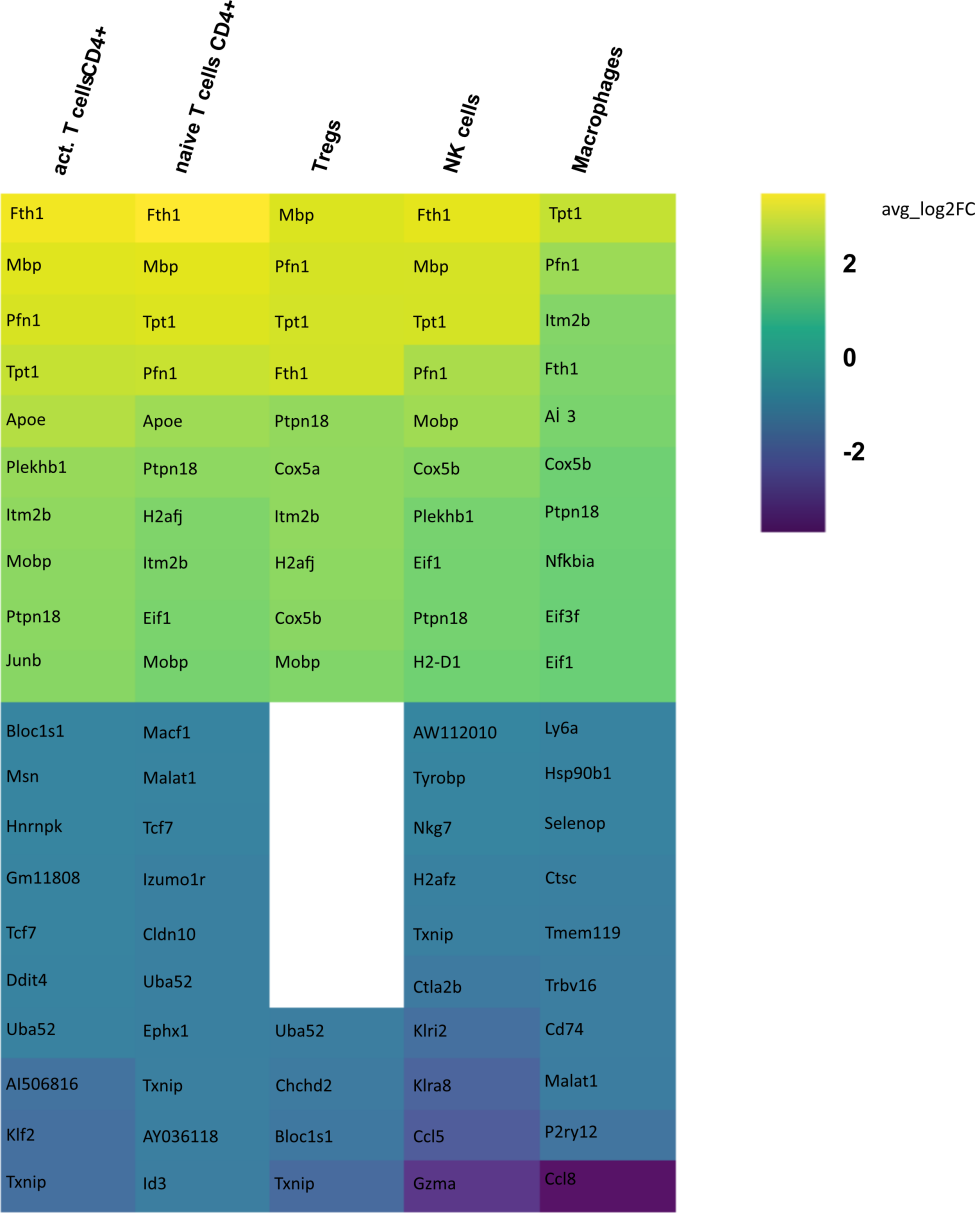
Heatmap representing differentially expressed genes (DEGs) between CSF from aCD20 treated and CSF from isotype treated mice for each cell type. Gene expression analysis was conducted only for cell counts >50. Thus, not all cell types are shown. The top 10 up- and downregulated genes are shown, ranked according to average log2 fold-change (avg_log 2FC). Positive avg_log 2FC represent upregulated genes in CSF from aCD20 treated mice, negative values correspond to downregulated genes.

In order to identify differences in gene expression in CSF between the aCD20 and isotype conditions, a differential gene expression analysis was conducted for each cell cluster within the respective tissue samples. Only those cell types were included in the analysis, where >50 cells were available in both conditions. **Figure 5** illustrates the 10 genes with highest fold change among the significant upregulated and downregulated genes in CSF from aCD20 treated mice compared to the control treated group. A full list of significant genes can be found in **Supplementary Table 1**.

**Figure 6:**
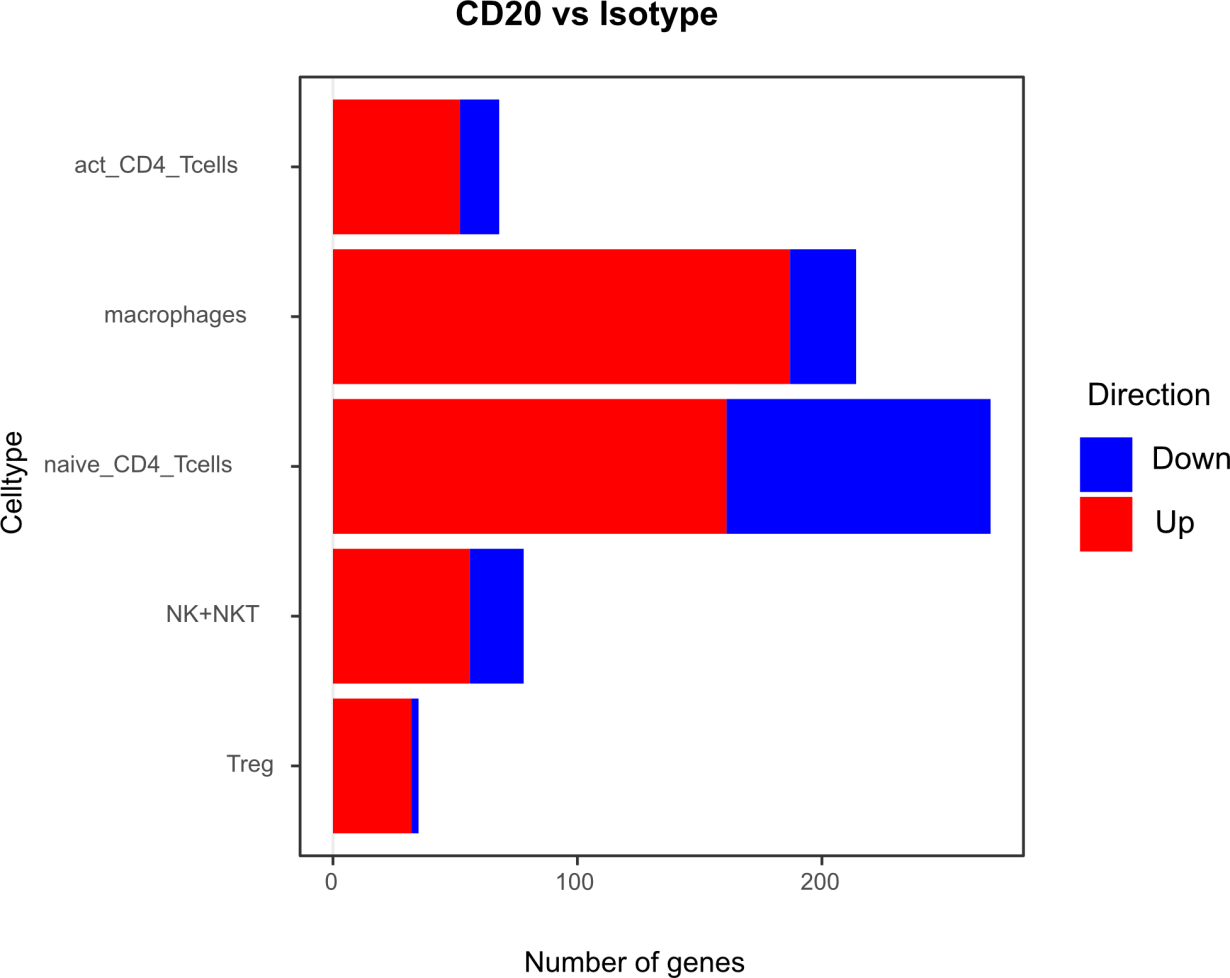
Number of genes that exhibit upregulation or downregulation in aCD20 treated mice compared to control treated mice.

Differential gene expression analysis showed that the majority of differentially regulated genes was upregulated in the aCD20 treated group in comparison to the control group (**Figure 6**). One of the most differentially expressed genes, upregulated in the aCD20 treated group in each of the examined cell types, was Ferritin Heavy Chain 1 (*Fth1)*, which has a major function in iron storage. Studies have demonstrated an upregulation of the *Fth1* gene in brain tissue of patients with MS lesions (22). However, an association with upregulated *Fth1* gene in immune cells of CSF is currently unknown. Another highly upregulated gene in all examined cell types was *Mbp*. The *Mbp* gene encodes for the myelin basic protein, which is a structural component of myelin sheath. Thus, Mbp expression in our data may represent contamination by myelin. However, according to Marty et al, Mbp expression is detected in T cells isolated from lymph nodes and spleen (23). Several variations in the *Mbp* gene have been associated with an increased susceptibility to developing MS and its clinical course (24). Furthermore, we observed a significant upregulation of the *Pfn1* gene in all immune cell populations. Even though no association with MS has been observed yet, studies have shown that excessive *Pfn1* activity can result in the immune system targeting the body’s own cells resulting in other autoimmune diseases (25). *Apoe* was among the top 10 upregulated genes in activated and naïve CD4+ T cells. *Apoe* exhibits notable immunomodulatory properties by attenuating immune activation through downregulation of immune stimulatory proteins on antigen-presenting cells. Multiple scientific investigations have established a potential association between *Apoe* and autoimmune conditions, including MS (26–28). Interestingly, both *Apoe* and *Mbp* also showed higher expression in mELT than in spleen and lymph nodes in many immune celltypes in our previous study, comparing mELT with secondary lymphoid organs (11). Macrophages in CSF of B cell depleted mice exhibited upregulated expression of *Atf3*. *Atf3* correlates with macrophages infiltration and plays a role in inflammation, cell division, and apoptosis (29). Additionally, we observed an upregulation of the *Junb* gene in immune cells from aCD20 treated mice. *Junb* has been identified as a significant contributor to the activation of T cells and the differentiation and proliferation of Tregs (30). In addition, several subunits of cytochrome c oxidase (*Cox5a*, *Cox5b*, *Cox8a*, *Cox6a1*), which play an important role in regulation of ATP synthesis via oxidative phosphorylation (OXPHOS) (31), were found to be upregulated after CD20 treatment in all studied cell types. Typically, resting lymphocytes generate energy through oxidative phosphorylation and fatty acid oxidation, whereas activated lymphocytes rapidly shift to glycolysis (32). Finally, also translation initiation factors including *Eif1*, and *Eif3f*, were upregulated by CD20 treatment in all studied cell types. Interestingly, another translation initiation factor has been found to play a role in MS development (33). aCD20 mAb treatment leads to an increased expression of proinflammatory chemokines CCL3 and CCL4 in macrophages, which can lead to the attraction of more macrophages and monocytes. In addition, also CXCL14 and CCRL2 were increased in aCD20 mAb-treated CSF samples.

While genes upregulated after aCD20 treatment show more consistency across the different cell types, there is more variability observed in downregulated genes between different cell types. Among the genes found to be downregulated in the CSF after aCD20 treatment was *Txnip*, which was downregulated in activated CD4+ T cells, naive CD4+ T cells, Tregs, and NK cells. The *Txnip* gene has been investigated for its role in inflammatory activation and its implication in neurodegenerative disorders (34). In NK cells, the *Gzma* gene is downregulated in CSF from aCD20 treated mice. The *Gzma* gene, also known as Granzyme A, encodes a serine protease enzyme that is primarily found in the granules of cytotoxic T lymphocytes (CTLs) and NK cells. *Gzma* has been associated with immune activation and promotion of inflammation (35). A downregulated gene in macrophages is the *Ccl8* (C-C Motif Chemokine Ligand 8) gene. *Ccl8* has been linked to a severe disease course in MS (36). Furthermore, *Ccl8* is also known for its role in the induction of inflammation and is highly expressed in tumor-associated macrophages (37).

Although some of the top downregulated genes seemed to be related to inflammation and some of the top upregulated genes are related to regulation of inflammation, overall, gene expression analyses comparing CSF immune cells of B cell depleted and non-depleted mice did not show a uniform pro- or anti-inflammatory pattern.

### The majority of differentially expressed genes were downregulated in mELT compared to CSF

**Figure 7:**
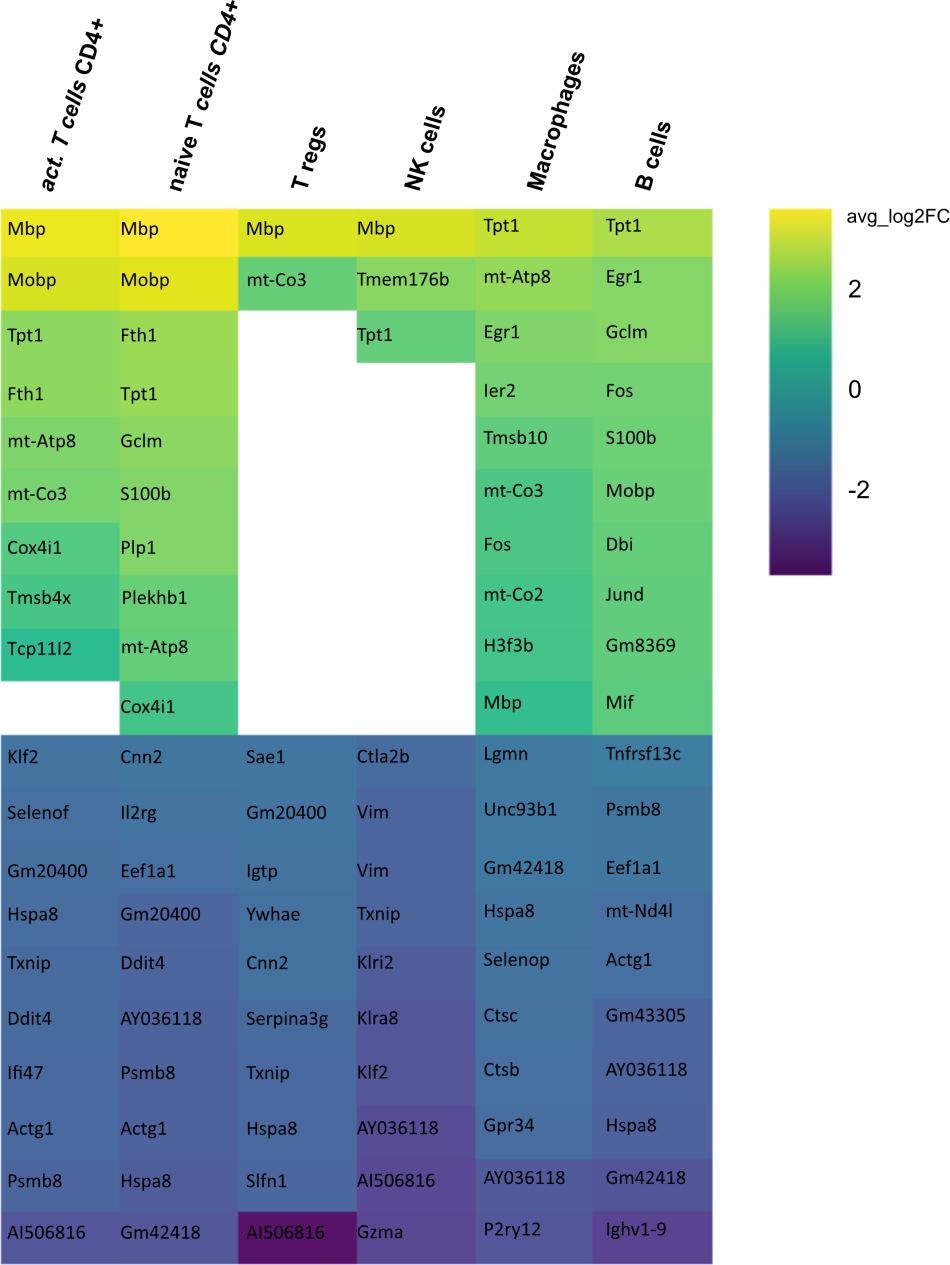
Heatmap representing differentially expressed genes (DEGs) between mELT from control treated and CSF from control treated mice for each cell type. Gene analysis was conducted only for cell counts >50. Thus, not all cell types are shown. The top 10 up- and downregulated genes in mELT from control treated mice when compared to CSF from control treated mice are shown. Genes were ranked according to average log2 fold-change (avg_log 2FC). Positive avg_log 2FC represent upregulated genes in mELT from control treated mice (green), negative values correspond to downregulated genes (blue).

**Figure 8:**
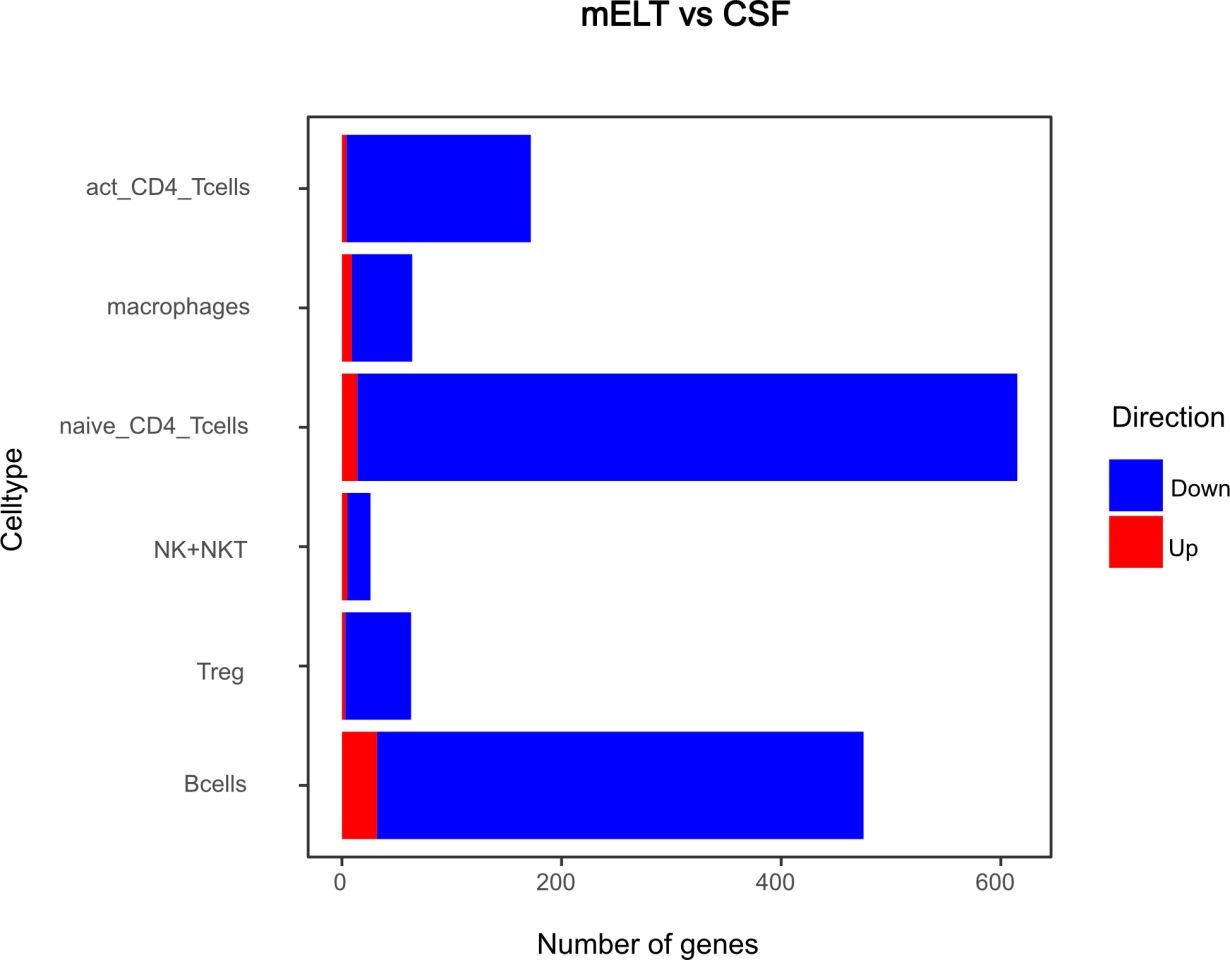
Number of genes that exhibit upregulation or downregulation in mELT in comparison to their expression levels in CSF.

Secondly, we compared the gene expression profiles of immune cells in mELT with CSF in control treated, B cell competent EAE mice. In mELT compared to CSF, overall, more genes were downregulated than upregulated (**Figures 7**, **8**). A full list of significant genes is in **Supplementary Table 2**.

However, there was an overlap between the top 10 upregulated genes when comparing mELT versus CSF in the control treatment condition (**Figure 7**) with CSF in aCD20 treatment versus CSF in control treatment (**Figure 5**). Among the top 10 upregulated genes were once again *Mbp*, *Mobp*, *Fth1*, and *Tpt1*. These genes have been consistently associated with crucial biological processes, including myelin formation (*Mbp*, *Mobp*), iron homeostasis (*Fth1*), and cellular stress response (*Tpt1*). The expression of *Mbp*, *Mobp* and *Plp1*, which is also among the top 10 upregulated genes in the naïve CD4+ T cell dataset, could possibly represent myelin contamination in mELT. In B cells, the expression of immediate early genes (IEGs) such as *Egr1*, *Jund*, and *Fos*, which are rapidly induced in response to antigen receptor or cytokine stimulation (38), exhibited higher levels of expression in mELT compared to CSF. Interestingly, we found IEG also to be higher expressed in mELT compared to lymph nodes and spleen in our previous study (11). It is noteworthy that one of the most upregulated genes in B cells was *Fos*. Previous studies have associated *Fos* with MS (39).

The majority of genes was downregulated in mELT compared to CSF, including the *Klf2* gene, also referred to as Krüppel-like factor 2, downregulated in activated CD4+ T cells and NK cells, naïve CD4+ T cells and B cells. *Klf2* is recognized as a negative regulator of EAE-induced neuroinflammation through the inhibition of pro-inflammatory factors. It has been demonstrated that the absence of *Klf2* exacerbates neurological dysfunction and neuroinflammation (40). Furthermore, in mELT of control-treated mice, there were additional downregulated genes that seem noteworthy - *Hspa8* (all cell types), and *Gzma* (NK cells). G*zma* has been introduced above. *Hspa8* belongs to the *Hsp70* family of chaperones and exhibits a protective potential in neurodegenerative diseases (41). Additionally, *Hsp70* serves as a marker for inflammatory processes in MS. It has been demonstrated that patients with MS have an elevated level of *Hsp70* in their serum. Specifically, clinically isolated syndrome and relapsing-remitting MS are associated with higher serum concentrations of *Hsp70* compared to progressive MS (42).

In summary, gene downregulation outweighed upregulation in control treated mELT compared to CSF – a finding that remains difficult to interpret.

### Pathway enrichment analysis suggests that B cell depletion from CSF has little effect on immune-related pathways in remaining cells

Pathway enrichment analysis was used to identify differentially regulated pathways among differentially expressed genes (both up and downregulated) from 1) the CSF in the aCD20 vs. control treated conditions and 2) B cell competent immune cells between mELT and CSF.

**Figure 9:**
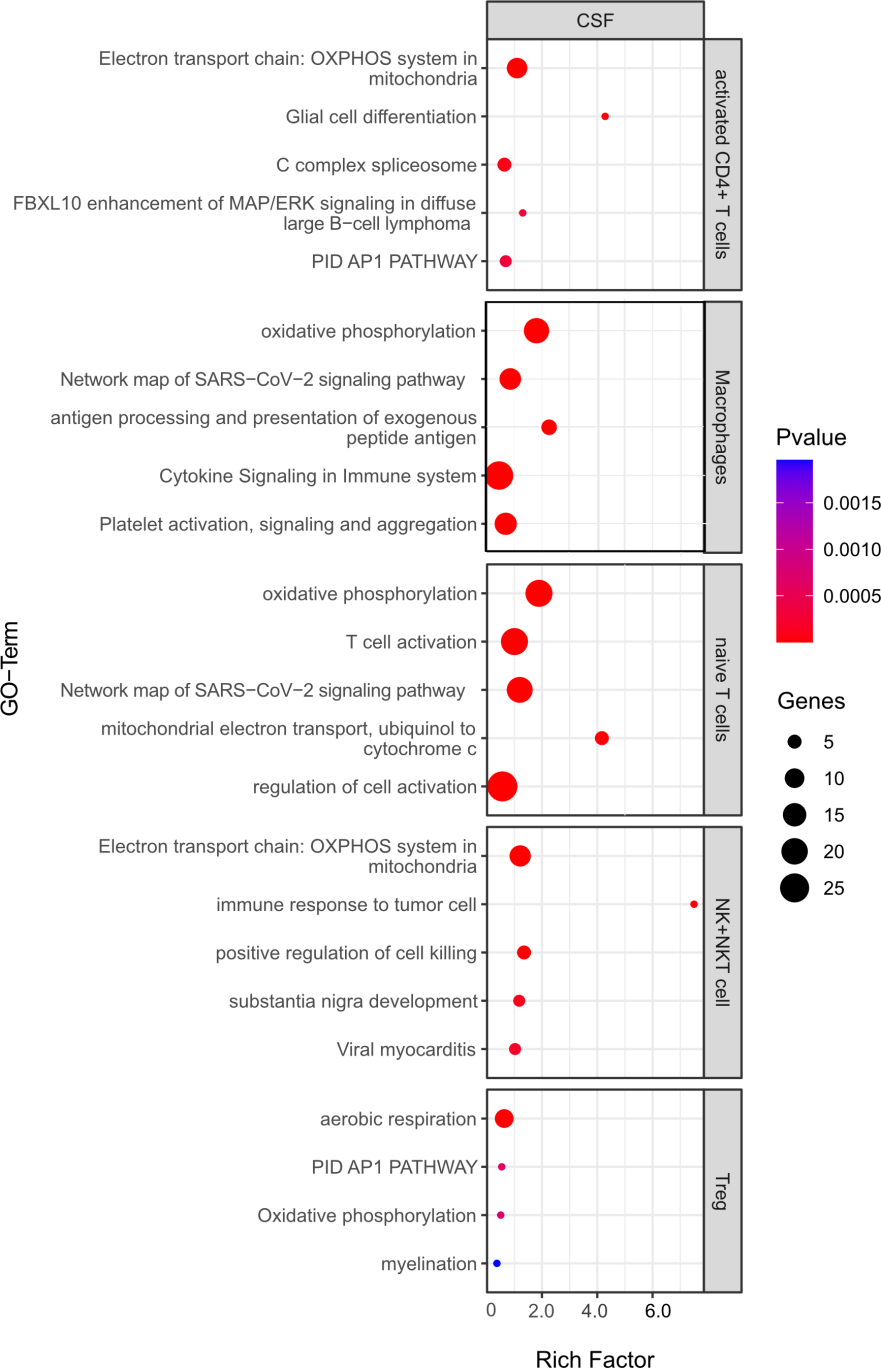
Functional enrichment analysis for genes differentially expressed between CSF from aCD20 treated mice and CSF from control treated mice using Metascape. The top 5 GO-terms are shown (For Tregs, only four pathways were significant). For similar gene ontology (GO) terms, only the top-ranked GO term was shown. The dot size reflects the number of genes enriched in the GO term and its color the corresponding statistical significance (p-value). Rich factor is the ratio of differentially expressed gene numbers annotated in this GO-term to all gene numbers annotated in this GO term.

For the first analysis, the top 5 pathways per cell type are shown in **Figure 9**. The most relevant seem to be T cell activation (in naïve T cells), antigen processing and presentation of exogenous peptide antigen and cytokine signaling in immune system (both macrophages). Moreover, oxidative phosphorylation, which is known to be important for many immune cell functions (32), was among the top pathways for all cell types. Yet, the majority of regulated pathways seem not to be primarily linked to inflammation. Overall, this analysis suggests that the absence of B cells in the CSF does not significantly change the inflammatory state of the remaining immune cells, whereas the phenotype (cell type composition) is clearly different.

**Figure 10:**
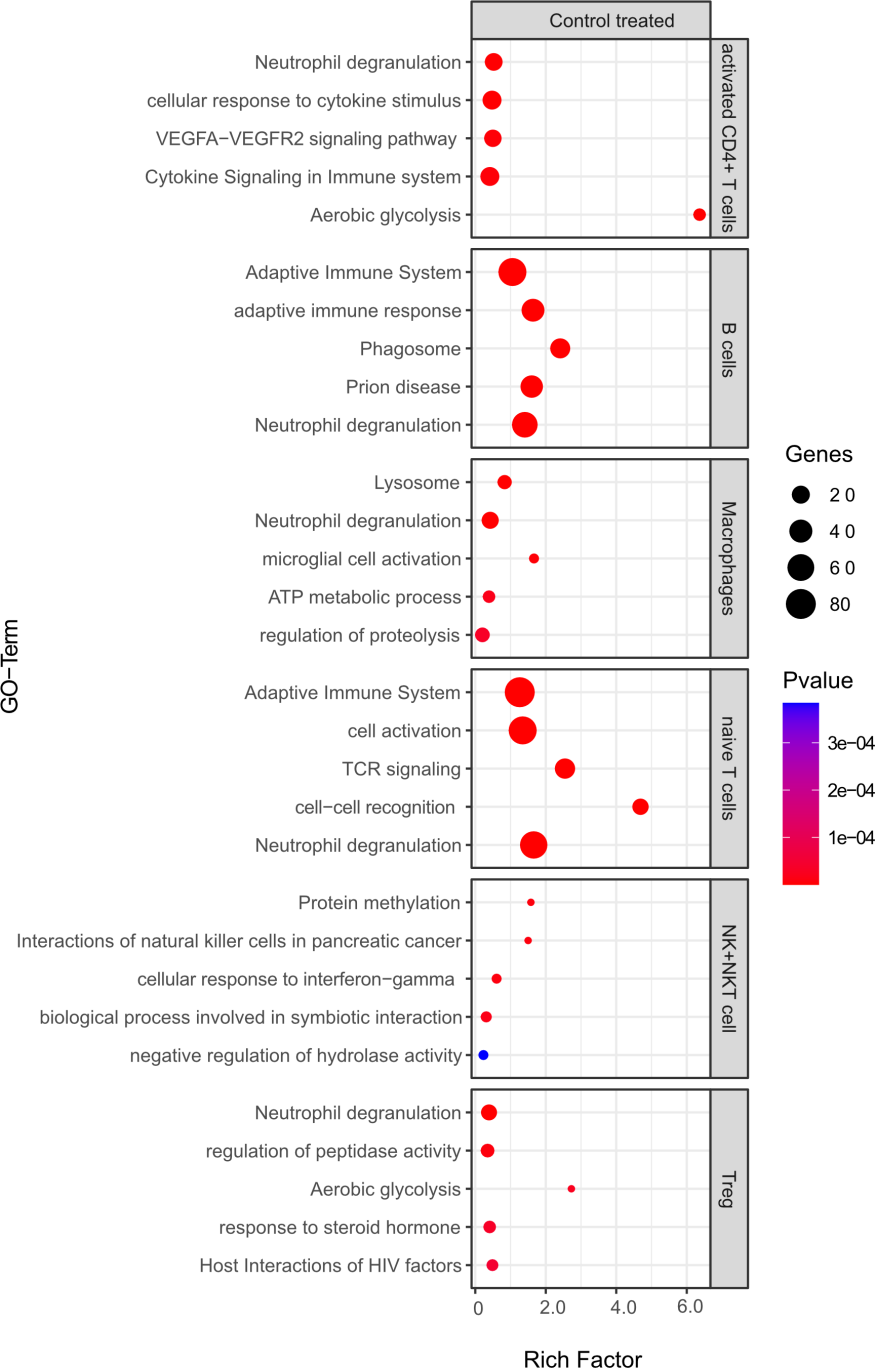
Functional enrichment analysis for genes differentially expressed between mELT from control treated mice and CSF from control treated mice using Metascape. The top 5 GO-terms are shown. For similar gene ontology (GO) terms, only the top-ranked GO term was shown. The dot size reflects the number of genes enriched in the GO term and its color the corresponding statistical significance (p-value). Rich factor is the ratio of differentially expressed gene numbers annotated in this GO term to all gene numbers annotated in this GO term.

Comparing B cell competent immune cells between mELT and CSF (**Figure 10**), we found an enrichment of relevant immune-related pathways, for example response to cytokine stimulus/signaling in activated CD4+ T cells, adaptive immune system response in B cells and naïve CD4+ T cells, and activation, cell-cell recognition and TCR signaling in naïve T cells.

## Discussion

Based on our previously published observation, that aCD20 mAbs efficiently deplete B cells from mELT in spontaneous EAE but do not prevent or inhibit mELT formation, we set out to compare mELT that contained B cells with mELT in which B cells were depleted by single cell RNA sequencing. Unfortunately, due to unresolved technical challenges, we were unable to obtain, process and analyze enough cells from mELT in aCD20 mAbs treated mice to generate meaningful results in this condition. However, sufficient data could be obtained from control treated (B cell competent) mELT and from the CSF of aCD20 mAbs treated and control treated mice. Thus, we addressed our second objective first: whether the CSF and mELT compartment are related. Our data suggest that the immune cell composition, and to some degree gene expression profile, in the CSF mirrors that in mELT in B cell competent spontaneous EAE mice. Both, CSF and mELT, are predominated by B and CD4+ T lymphocytes. While other studies have highlighted certain aspects of the cellular composition of mELT, mostly based on morphological and immunohistochemical features (6, 16), our previous study has established mELT as a tertiary lymphoid organ closely resembling secondary lymphoid organs (11). Our current report is the first to study murine CSF in EAE by single cell RNA sequencing and comparing it with mELT. The cellular composition and immune phenotype of CSF cells in MS patients and healthy individuals has been studied quite intensively. While the CSF of healthy individuals contains < 5 leukocytes per µl, which are predominantly CD4+ T lymphocytes and fewer monocytes (43), CSF in MS patients may feature mild pleocytosis and a substantial increase in B cells (44). The cellular composition of murine CSF has not been described, to our knowledge. Our data suggests that CSF cells may serve as a surrogate for mELT in our model.

Analyzing the effects of aCD20 mAbs on the CSF compartment revealed, as expected, near complete depletion of B cells. We have previously demonstrated virtually complete B cell depletion from mELT by aCD20 mAbs in the same model using immunohistochemistry (16). Another study demonstrated that aCD20 mAbs could deplete B cells from the meninges, in a model that does not feature mELT (45). Interestingly, we observed a concomitant decrease of naïve CD4+ T cells in the absence of B cells in the CSF. It seems possible that a fraction of CSF T cells, that expressed CD20 on their cell surface, may directly be depleted by aCD20 mAbs (46). Monocytes, plasma cells and granulocytes were also decreased. In contrast, macrophages were markedly increased. This increase exceeded a mere relative, compensatory increase of other cell types in the absence of B cells but seems to be a distinct finding. Our previous study has suggested that macrophages in mELT may stimulate the recruitment of other immune cells to the inflamed CNS (11). We speculate that similar mechanisms may apply to the CSF. Peripheral blood CD11b+ monocytes/macrophages in B cell depleted patients have a more pro-inflammatory phenotype compared to B cell competent patients (47). Possibly, this altered phenotype favors accumulation of those myeloid cells in the CSF. In line with our data, studies have demonstrated that B cell depletion by aCD20 mAbs like rituximab and ocrelizumab in MS patients led to a strong reduction of CSF B cells and, to a lesser degree, T cells (48–50). To our knowledge, no data regarding CSF macrophages in B cell depleted CSF has been published.

Gene expression and pathway enrichment analyses comparing B cell competent mELT with CSF immune cells on the one hand and aCD20 mAbs versus control treated CSF on the other hand did not reveal striking differences with regard to immune cell phenotypes. Despite minor variations, no overarching theme directing any of the conditions in a specific way, either pro- or anti-inflammatory, could be identified. Given the remarkable efficacy of B cell depleting mAbs in treatment of MS, one could have expected a less inflammatory profile in B cell depleted CSF. This may be a limitation of the model we used, as it does not respond to aCD20 mAbs clinically.

This study faced other challenges and limitations. We discussed above how we were unable to obtain sufficient cells from B cell depleted mELT. We did not expect this challenge, since we knew from our previous study that mELT forms under B cell depleted conditions. However, B cell depleted mELT is less dense and structured. Thus, we speculate that this may have caused our difficulties in dissecting enough cells. Another major limitation was that we had to exclude one mouse from the aCD20 treated group due to insufficient B cell depletion after aCD20 mAbs treatment, and CSF cells could not be obtained from one control treated mouse. This prevented statistical analyses. For cost reasons, this study was planned with 3 mice per group, which is also a limitation to the study. Another limitation of our study is that we isolated immune cells from the entire meninges. Therefore, it is possible that, next to immune cells from mELT, our mELT samples may also contain immune cells present in the meningeal compartment outside of mELT.

In summary, one major finding of our study was that the immune cells in the CSF closely resemble those in mELT in 2D2xTh EAE mice. This included particularly the cellular composition and, to some degree, gene expression profiles. Given the relatively easy accessibility of CSF in MS patients, it would be helpful to be able to use CSF as a surrogate for mELT in MS. Future studies will have to verify this finding in humans. Another major finding was the increase of macrophages in the CSF that we observed when B cells were depleted. This also needs to be investigated in CSF of aCD20 mAb treated MS patients. While PIRA remains a challenge in the B cell depletion treatment era in MS, understanding the essential mechanisms of smoldering CNS inflammation, including in mELT and the CSF, is of great importance. It remains to be seen if Bruton’s tyrosine kinase (BTK) inhibitors or other novel agents will be able to stop smoldering inflammation in the CNS, e.g. by inhibiting the formation of mELT.

## Data availability statement

The names of the repository/repositories and accession number(s) can be found below: GEO repository, https://www.ncbi.nlm.nih.gov/geo/, GSE181506.

## Ethics statement

The animal study was approved by standing committee for experimentation with laboratory animals of the administration of Upper Bavaria (ROB-55.2-2532.Vet_02-16-100). The study was conducted in accordance with the local legislation and institutional requirements.

## Author contributions

TG: Conceptualization, Data curation, Formal analysis, Validation, Writing – original draft, Investigation, Visualization. JD: Data curation, Formal analysis, Investigation, Validation, Visualization, Supervision, Writing – review & editing. VF: Formal analysis, Investigation, Supervision, Validation, Writing – review & editing, Conceptualization. GL: Formal analysis, Writing – review & editing, Data curation, Software, Visualization. RB: Formal analysis, Writing – review & editing, Investigation. KL-H: Formal analysis, Writing – review & editing, Conceptualization, Data curation, Funding acquisition, Resources, Supervision, Validation, Writing – original draft.
